# Association Between Oral Health Complaints and Nutritional Status among Patients with Cancer: A Study Utilising the Mini Nutritional Assessment Short Form

**DOI:** 10.3290/j.ohpd.c_1827

**Published:** 2025-01-23

**Authors:** Sachin Naik, Sajith Vellappally, Mohammed Alateek, Abdulaziz Abdullah Al Kheraif, Mohammed Alghamdi, Sukumaran Anil

**Affiliations:** a Sachin Naik Associate Professor, Dental Health Department, College of Applied Medical Sciences, King Saud University, P.O Box. 10219, Riyadh, 11433, Saudi Arabia. Conceptualisation, methodology, writing (original draft preparation).; b Sajith Vellappally Associate Professor, Dental Health Department, College of Applied Medical Sciences, King Saud University, P.O Box. 10219, Riyadh, 11433, Saudi Arabia. Conceptualisation, methodology, writing (original draft preparation), resources.; c Mohammed Alateek Dental University Hospital, King Saud University, Medical City, Riyadh, Saudi Arabia. Conceptualisation, formal analysis and investigation.; d Abdulaziz Abdullah Al Kheraif Professor, Dental Health Department, College of Applied Medical Sciences, King Saud University, P.O Box. 10219, Riyadh, 11433, Saudi Arabia. Methodology, writing (review and editing), funding acquisition, supervision.; e Mohammed Alghamdi Director, Oncology Centre, King Khalid University Hospital, King Saud University Medical City, Riyadh, 11433, Saudi Arabia. Methodology, writing (review and editing).; f Sukumaran Anil Pushpagiri Institute of Medical Sciences and Research Centre,Thiruvalla,689101, Kerala, India. Methodology, writing (review and editing).

**Keywords:** BMI, cancer, malnutrition, mini nutritional assessment, oral health

## Abstract

**Purpose:**

Oral health problems in patients with cancer can substantially affect their quality of life, treatment outcomes, and overall nutritional well-being. This study investigated the relationship between nutritional status and self-reported oral health complaints in patients with cancer.

**Materials and Methods:**

A cross-sectional study was conducted among patients with cancer at the King Saud University Medical City Oncology Center in Riyadh, Saudi Arabia. Patients’ nutritional status was assessed using the mini nutritional assessment short form (MNA-SF), and self-reported oral health problems were documented. Data were analysed using the Chi-square test and multinomial logistic regression.

**Results:**

This study included 200 participants who completed both the MNA-SF assessment and self-reported their oral health complaints. Common oral health problems included xerostomia (81%), bleeding gums (60.5%), toothaches (35%), and mouth ulcers (24%). Malnourished individuals reported higher rates of all oral complaints, with 54% of them experiencing bleeding gums, 50% reporting toothaches, and 54% experiencing speech problems. Notably, 71% of malnourished patients reported mouth ulcers (P < 0.05). Regression analysis revealed a statistically significant association (P < 0.05) between xerostomia and the ‘At risk of malnutrition’ group, with an odds ratio of 1.004 (95% confidence interval [CI]: 0.411–2.449). In the ‘Malnourished’ category, mouth ulcers showed a statistically significant association (P < 0.05) with an odds ratio of 1.402 (95% CI: 0.409–4.800).

**Conclusion:**

Our findings highlighted statistically significant correlations between nutritional status, as assessed using the MNA-SF, and oral health complaints in patients with cancer. Well-nourished individuals reported fewer oral complaints, whereas malnourished patients reported a higher prevalence of oral health issues.

Oral health is critical for overall well-being, particularly in patients with cancer undergoing treatment. In Saudi Arabia, the prevalence of cancer is rising, with projections indicating a significant increase of 116.7% in new cases by 2040.^1^ Concurrently, malnutrition poses a substantial health concern among patients with cancer, with studies showing that up to 26% of such patients may be malnourished.^3^ Patients with cancer frequently experience a range of oral health problems, creating a complex relationship between cancer, nutrition, and oral health. These oral health challenges can significantly affect a patient‘s quality of life (QoL), treatment outcomes, and overall nutritional well-being.^21,40^ The bidirectional nature of this relationship is increasingly recognised; oral health issues can lead to malnutrition, whereas poor dietary status can exacerbate oral health problems.

Malnutrition in patients with cancer often results from a combination of disease-related factors and side effects of treatment. These include decreased nutrient consumption, loss of appetite, and changes in taste, often accompanied by psychological factors such as fear, depression, and anxiety.^16^ Certain nutrients, particularly vitamins and proteins, are crucial in maintaining oral health by supporting tissue repair and enhancing the immune system’s capacity to fight oral infections.^33^ Conversely, nutritional deficiencies can worsen oral health problems and aggravate pre-existing conditions.^24^

Patients with cancer frequently experience debilitating symptoms such as fatigue, anorexia, and pain, along with oral health issues, including dry mouth (xerostomia), candidiasis, dysphagia, ulcers, and mucositis. These oral problems severely impact QoL by affecting essential functions such as eating and speaking and can exacerbate psychological distress.^15^ Studies have shown strong associations between oral health, nutrition, and QoL in patients with cancer.^5,9,11,29,37^

Research indicates that integrating oral healthcare into cancer care can enhance the ability to eat, speak, and swallow and improve nutritional status. Optimising nutritional status before, during, and after cancer treatment is recommended to improve treatment tolerance.^5^

Cancer treatment further complicates the relationship between nutritional status and oral health in patients with cancer. Chemotherapy and radiation therapy can contribute to oral complications such as mucositis, xerostomia, alterations in salivary composition, and changes in taste perception.^24,28^ These complications can significantly impact a patient’s ability to eat, speak, and maintain oral hygiene.^34^ For instance, mucositis and dysphagia can lead to reduced food intake, potentially resulting in malnutrition.^7^ Consequently, malnutrition can weaken the immune system and impair wound healing, making patients more susceptible to oral infections and ulcers.^7^ This creates a potential cycle where oral health issues lead to poor nutrition, which can worsen oral health.

Although some interventional studies have shown promising results, such as the potential of nutritional supplements to reduce the severity of oral mucositis and accelerate healing, research in this area remains limited.^24,27,42^ Further studies are required to develop effective interventions to address the complex relationships between nutrition, oral health, and cancer.

This study aimed to analyse the correlation between nutritional status and self-reported oral health complaints among patients with cancer, focusing on specific issues such as bleeding gums, toothaches, mouth ulcers, bruxism, xerostomia, and speech-related difficulties. By exploring these associations, we hope to contribute to the progress and development of integrated approaches to cancer care that concurrently address nutritional and oral health needs.

## MATERIALS AND METHODS

### Study Design and Participants

**Fig 1:**
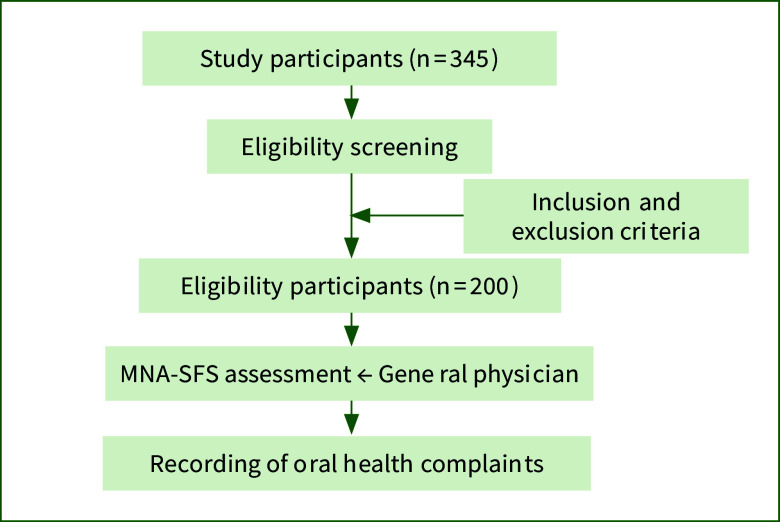
Flowchart of the study methodology.

This cross-sectional study focused on individuals diagnosed with cancer who were recruited from King Khalid University Hospital, King Saud University Medical City, Riyadh, Saudi Arabia. Data were collected between December 2023 and February 2024 (Fig 1). Potential participants were identified through their medical records and oncologist referrals. Eligible patients were contacted, provided with the study information, and invited to participate. Patients of interest underwent a screening process to confirm their eligibility.

The study included patients aged 18 years or older, with a cancer diagnosis or undergoing treatment, who provided informed consent either personally or through a caregiver. The exclusion criteria included patients with severe cognitive impairment, those with terminal illness, those who declined to provide informed consent, and pregnant women. The sample size for this study was calculated using G*Power software (version 3.1.9.7). We based our calculation on previous literature examining the prevalence of malnutrition in patients with cancer.^2^ Assuming a moderate effect size (Cohen’s w = 0.3), an alpha level of 0.05, and a desired power of 0.80; the minimum required sample size was estimated to be 196 participants. To account for potential dropouts or incomplete data, we recruited 200 participants.^10^ This sample size was deemed adequate to detect clinically meaningful differences in oral health complaints between nutritional status groups, while also allowing for potential subgroup analyses.

Data were collected using a survey form comprising two main sections: demographic information and a self-reported oral health complaint questionnaire. Oral health complaints were selected based on insights from prior literature,^14,22,40^ with patients personally recording their complaints to ensure that the data reflected their direct experiences.

Marital status data were collected to understand the role of social and emotional support in managing the side effects of treatment, including nutritional and oral health complaints.

Nutritional status was assessed using the mini nutritional assessment short form (MNA-SF), a validated tool comprising components across four key areas^18^: anthropometric measurements (height, weight, body mass index [BMI]), general health indicators (medical history, medication use, mobility), dietary habits and nutritional intake, and subjective health perceptions.

A pilot study was conducted over 2 weeks with 5% of the study sample to assess its feasibility and practicality. Data were collected using Google Forms, which allowed participants to complete the online survey. A team of experienced physicians and nursing staff assessed participants’ nutritional status.

### Ethical Considerations

This study was approved by the Ethics Committee of King Saud University (approval number: 23/0910/IRB).

### Statistical Analysis

SPSS version 27.0 (Armonk, NY: IBM Corp) was used for all data analyses. Qualitative data were analysed using frequency distributions. The relationship between oral health complaints and MNA-SF scores was analysed using the Chi-squared test. Quantitative data, including BMI, were analysed using descriptive statistics.

MNA-SF scores were classified into three categories: well-nourished (≥ 24), at risk of malnutrition (17 to < 23.5), and malnourished (< 17).

Multinomial logistic regression was conducted to explore the relationship between independent variables (oral health complaints and cancer treatment type) and dependent variables (MNA-SF nutritional status). Statistical significance was set at a P value of less than 0.05.

## RESULTS

Table 1 presents the participants’ characteristics. Men comprised 56.5% of the sample, whereas women comprised 43.5%. The 45–64 years age group represented approximately 48% of the participants, with 30% aged 65 years or older. Colon cancer was the most prevalent type of cancer (34.5%), followed by breast cancer (32%). The remaining 28.5% of patients had other types of cancer. The cancer treatments included chemotherapy (90%), radiation (33.5%), and surgery (33.5%). The participants exhibited statistically significant variability in BMI, with an average of 28 (standard deviation: ±17.06).

**Fig 2:**
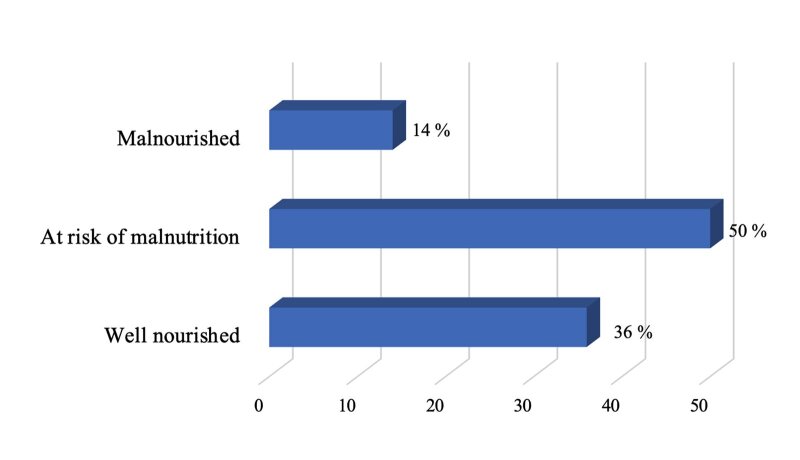
Nutritional status of the study participants according to mini nutritional assessment short-form scores.

Among the oral health complaints, 60.5% of study participants reported bleeding gums, 35% reported toothache, 24% had mouth ulcers, and 4% had bruxism (Table 2). Most of the respondents (81%) had xerostomia. Only 5% of the participants had problems affecting their speech. Approximately 86% of the participants reported satisfactory food intake, whereas 14% expressed concerns. Figure 2 indicates that 36% of participants were well-nourished, 50% were at risk of malnutrition, and 14% were malnourished.

Table 3 shows that over the past 3 months, 29% of people lost more than 3 kg of weight, raising weight loss concerns. Moreover, 95% of participants live independently without a bed or chair. Approximately 53.5% of patients had mild dementia. Regarding protein intake, 81.5% of participants scored 1.5, indicating satisfactory protein intake. A statistically significant proportion (76.5%) reported consuming fruits and vegetables daily. Furthermore, most participants (67%) perceived their health to be better than others, and 75% reported no nutritional difficulties. Notably, approximately 73.5% of the participants had BMIs exceeding 23, indicating a tendency towards higher body weight.

**Fig 3:**
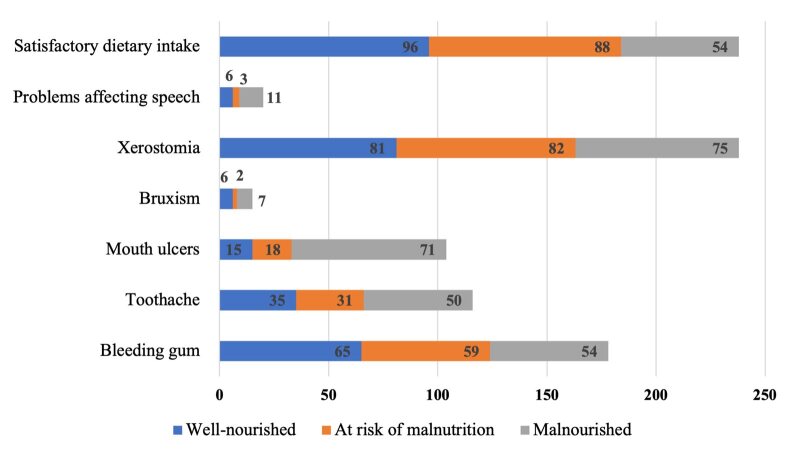
Percentage of oral health complaints in patients with cancer, based on nutritional status.

Figure 3 illustrates the relationship between oral health complaints and nutritional status based on MNA-SF scores. Well-nourished individuals exhibited lower rates of toothaches (35%), problems affecting speech (6%), and bleeding gums (65%), as determined by the MNA-SF assessment.

Table 4 shows statistically significant differences in satisfactory dietary intake among the different nutritional status groups (P <0.001). Malnourished participants reported higher rates of various oral complaints, including bleeding gums (59%), toothaches (31%), and speech problems (82%). Additionally, xerostomia was reported in 82% of the malnourished participants. Malnourished individuals also had significantly higher rates of mouth ulcers, with 71% affected (P <0.03).

Our findings indicate that chemotherapy may contribute to an increase in oral health complaints, specifically bleeding gums, toothaches, and speech-related problems. Among the observed complaints, 83% reported experiencing xerostomia, whereas 17% did not encounter such issues (P = 0.04). This suggests a statistically significant association between chemotherapy and xerostomia.

In the BMI category of 23 or greater, 60% of individuals reported bleeding gums, 40% reported toothache, and 28% reported problems affecting speech. The occurrence of oral complaints varied across BMI categories. Individuals who reported weight loss exceeding 3 kg exhibited a notable association with problems affecting speech, with a higher percentage (24%) reporting this issue compared with those without weight loss (13%) (P = 0.02). Additionally, these patients demonstrated higher rates of toothaches (40%) and bleeding gums (50%) than the general population. According to the MNA-SF, people who took more than three prescription medications daily reported a higher percentage of toothache (75%) compared with those in the ‘No’ category (65%) (P > 0.05).

Individuals with severe depression showed a statistically significant association with problems affecting speech, with a higher percentage (83%) than in those without severe depression (17%) (P = 0.02). Severe depression was also statistically significant associated with a higher prevalence of xerostomia, with 83% reporting xerostomia compared with 17% without severe depression (P = 0.03).

The results of the multinomial regression analysis for the malnutrition categories are presented in Table 5. In the ‘At risk of malnutrition’ category, chemotherapy revealed a marginally significant association (OR: 1.335; 95% confidence interval [CI]: 0.246–2.841; P = 0.07); xerostomia exhibited a statistically significant association (OR: 1.004; 95% CI: 0.411–2.449; P < 0.05); and a satisfactory diet was also statistically significantly associated (OR: 0.296; 95% CI: 0.076–1.150; P <0.05). In the ‘Malnourished’ category: chemotherapy was statistically significantly associated (OR: 1.517; 95% CI: 0.096–2.769; P < 0.05); mouth ulcers showed a statistically significant association (OR: 1.402; 95% CI: 0.409–4.800; P < 0.05); xerostomia showed a statistically significant association (OR: 1.819; 95% CI: 0.232–2.895; P < 0.05); and a satisfactory dietary intake showed a substantial association (OR: 0.05; 95% CI: 0.013–0.242; P < 0.05). These results indicate that the impact of xerostomia and mouth ulcers on nutrition may be influenced by oral health problems, leading to a connection between chemotherapy, oral health, and nutrition.

## DISCUSSION

The nutritional status and oral health of patients with cancer are crucial for their overall well-being and treatment outcomes. This study explored the relationship between nutritional status and self-reported oral health complaints in patients with cancer and revealed statistically significant associations that underscore the complex interplay between cancer, nutrition, and oral health. Using the MNA-SF, we found that well-nourished patients had fewer oral health complaints, emphasising the link between oral health and nutrition. This aligns with previous studies that have highlighted the connection between nutrition, dental health, and cancer prognosis.^4,8,26,35^ According to Polański et al, cancer and its treatments can decrease food intake, alter metabolism, and cause malnutrition. Our study corroborates these findings and demonstrates the prevalence of malnutrition in patients with cancer.^31^

A key finding of our study was the statistically significant higher prevalence of mouth ulcers among malnourished individuals identified using the MNA-SF. This aligns with previous research that established a connection between vitamin deficiencies, malnutrition, and problems with oral mucosal tissues.^12^ Liu et al^25^ emphasised the role of nutrition in improving the survival rates of individuals with oral cancer. Higher consumption of fruits, vegetables, and proteins benefits oral health.^28,34^

Patients with cancer often take high-sugar supplements to maintain their energy levels during treatment. However, these supplements, combined with reduced oral hygiene due to treatment-related pain, can increase the risk of oral diseases, such as mucositis, candidiasis, and dental caries. Hence, proper oral care is essential for mitigating these risks.^39^

Our study supports previous findings by Epstein et al, who identified common oral health problems in patients with cancer, including xerostomia, oral mucositis, altered taste, and difficulty swallowing.^13^ Oral mucositis, affecting 40–70% of patients undergoing chemotherapy and radiotherapy, emerged as a critical factor in this relationship.^32^ Mucositis-associated ulcers can cause intense pain and swallowing difficulties, potentially leading to malnutrition. Conversely, malnutrition can weaken the immune system, making patients more susceptible to severe mucositis and other oral infections.^32,34^ Xerostomia, a common side effect of cancer therapy, was found to lead to dysphagia, reduced food intake, and potentially cause malnutrition and weight loss. It impairs chewing and swallowing, necessitating dietary modifications and saliva substitutes for management.^32^

Our study found that well-nourished individuals across all BMI categories had fewer oral health complaints, aligning with previous research.^23^ Participants who experienced substantial weight loss showed a higher prevalence of bleeding gums and toothaches, highlighting the impact of weight loss on oral health deterioration. This suggests that achieving better overall health outcomes necessitates integrated healthcare approaches that address nutritional deficiencies and include weight management strategies.^30^ In an earlier study, researchers extensively examined dietary status as a predictor of the length of stay (LOS) among patients with cancer, alongside dental health. A thorough review was conducted regarding nutritional status and its relationship with LOS in patients with cancer, highlighting the importance of using standardised measurements across patient demographics and study designs.^19^ The study emphasised the high prevalence of malnutrition among patients with gastrointestinal cancer, and stressed the importance of timely nutrition education and professional care.^41^

The sstatistically significant findings of this study revealed that individuals who consumed more than three prescription drugs daily and experienced frequent malnutrition were more prone to bleeding gums and toothaches.

Our research also delved into psychological aspects, noting that severe depression demonstrated a notable association with problems affecting speech. This aligns with previous studies that emphasised the psychological impact of oral health problems on patients with cancer and the correlation between psychological symptoms and self-reported oral health.^20^ These findings underscore the importance of adopting a holistic approach with comprehensive care integrating oral hygiene, nutrition, and emotional support for patients with cancer, in order to tackle medication-related nutritional challenges and their repercussions on oral hygiene.^27^

While our study did not capture data on radiotherapy, cancer staging, medications, and systemic diseases, previous research has suggested that these factors can significantly affect oral health and nutrition in patients with cancer. Radiotherapy, especially in the head and neck region, is known to cause severe mucositis and xerostomia.^38^ Moreover, advanced cancer stages are often associated with higher rates of malnutrition.^17^ Additionally, certain medications, such as some chemotherapy agents, can directly affect oral health and appetite.^6^ Furthermore, comorbid systemic diseases such as diabetes can complicate both nutritional status and oral health.^36^

### Limitations

This study had a few limitations. First, the cross-sectional design limited the establishment of causality between nutritional status and oral health complaints; hence, longitudinal studies are required to provide more robust evidence. Second, generalizability may be limited because the participants were enrolled from a single oncology centre. Third, there was a lack of detailed information on radiotherapy treatment, cancer staging, medication regimens, and comorbid systemic diseases, which could substantially affect nutrition and oral health and potentially confound our results. Fourth, although the Mini MNA-SF is commonly used, its primary focus on the elderly may limit its applicability to younger patients with cancer. The reliance on self-reported oral health complaints may introduce recall bias, and the study’s emphasis on specific elements of oral health could make it challenging to obtain a comprehensive overview. Finally, this study did not account for potential differences in oral health complaints across various cancer types or stages. These limitations highlight the need for future research to address these gaps, particularly studies that include more detailed clinical information and employ objective oral health assessments.

## CONCLUSION

This study demonstrated a statistically significant relationship between nutritional status, as measured by the MNA-SF, and oral health complaints in patients with cancer. Well-nourished individuals exhibited lower rates of oral health issues, whereas malnourished individuals and those at risk of malnutrition reported a higher prevalence of oral health complaints. These findings underscore the broad impact of nutritional status on oral health in patients with cancer. Moreover, they highlight the need for a comprehensive approach to cancer care that integrates nutritional counselling, oral health assessments, and preventive measures alongside traditional treatment modalities. Further research is required to study the association between nutrition and oral health in patients with cancer. Interventional studies are required to determine the effectiveness of nutritional and oral health interventions in this population. Such research could inform the development of more targeted and effective care protocols for patients with cancer, potentially improving their overall treatment outcomes and well-being.

### Data Availability

The data supporting the findings of this study are available from the corresponding author upon reasonable request.

### Acknowledgements

This study was supported by Researchers Supporting Project number (RSP2024R31), King Saud University, Riyadh, Saudi Arabia.

### Conflicts of Interest

The authors declare no conflicts of interest.

### Consent to Participate

Informed consent was obtained from all individual participants included in the study.
